# Epikeratophakia for Keratoconus: A Case Report with 30 Years of Follow-Up

**DOI:** 10.1155/2023/9919057

**Published:** 2023-11-08

**Authors:** Takashi Miyai, Tetsuya Toyono, Hitoha Ishii, Kohdai Kitamoto, Yukako Taketani, Takashi Ono, Makoto Aihara, Kazunori Miyata

**Affiliations:** ^1^Department of Ophthalmology, University of Tokyo Graduate School of Medicine, Tokyo, Japan; ^2^Miyata Eye Hospital, Miyakonojo, Japan

## Abstract

**Background:**

Epikeratophakia is a refractive surgical procedure used to correct aphakic eyes, hyperopia, and keratoconus and is often performed in children. In this report, we present the long-term effects of epikeratophakia on the progression of keratoconus in a patient who underwent surgery. *Case Presentation*. The patient was a 17-year-old boy with keratoconus who had difficulty wearing hard contact lenses. As a solution, he underwent right eye epikeratophakia with a plano-powered lenticule. We followed up the patient for 30 years. Although the progression of keratoconus ceased in the operated eye, it continued in the nonoperated left eye and resulted in acute hydrops 9 years and 10 months after surgery. Subsequently, 20 years after the operation, anterior-segment optical coherence tomography was performed, which revealed that the progression of keratoconus had been interrupted in the right eye but had continued in the left eye, as evidenced by the parameters of the average and maximum keratometry and thinnest corneal thickness.

**Conclusions:**

Herein, we reported the longest follow-up to date of a case of keratoconus, in which one eye was treated with epikeratophakia. The progression of keratoconus was halted in the treated eye but continued in the nonoperated contralateral eye.

## 1. Introduction

Epikeratophakia is a refractive surgical procedure in which corneal lenticule tissue from a donor is sutured into the patient's cornea. This procedure is used to correct aphakic eyes, hyperopia, and keratoconus [1–6]. Refractive correction is achieved by transplanting a corneal lamellar disk without corneal endothelium, which helps suppress corneal graft rejection. Epikeratophakia is often performed in children. Its outcomes, that is, demonstration of adequate correction of refractive errors and safety within 10 years, have been primarily evaluated [4].

Herein, we present a case of keratoconus that was treated with unilateral epikeratophakia and followed up for 30 years. To the best of our knowledge, this is the longest ever reported follow-up period for keratoconus. Although the progression of keratoconus halted in the treated eye, it continued in the nonoperated contralateral eye, which led to an episode of acute hydrops.

## 2. Case Presentation

The patient was a 17-year-old boy with keratoconus who was diagnosed using a placid disk examination. He underwent epikeratophakia in the right eye owing to the difficulty in wearing hard contact lenses (HCLs). Preoperative visual acuity was RV = 0.02 (0.05×−4.0 D = cyl − 9.0 DA × 80°) (0.4 × HCL) and Vs = 0.2 (0.8×−11.0 D = cyl − 3.0 DA × 130°) (1.0 × HCL).

Kerato patch (power, +0.0 D; *K*-value, 0.0 D; AMO) was used as a lenticule. After epithelial removal, a 0.2 mm notch was made using the 7.5 mm Hessburg-Barron vacuum trephine, and a groove was made using the Katzin corneal transplant scissors (Storz). The lenticule was sutured to the host cornea using 16 single sutures with a 10-0 nylon thread. Two months after surgery, all threads were removed, and HCLs could be worn (RV = 0.1 (0.4×−2.5 D = cyl − 6.0 DA × 145°)). The postoperative course of the right eye was favorable. Four years and 10 months after surgery, visual acuity was as follows: Vd = 1.0 × HCL and Vs = 0.9 × HCL × −3.5 D. According to the anterior segment findings, no significant corneal protrusion was observed in the operated eye, and mild keratoconus was observed in the left eye.

**Figure 1:**
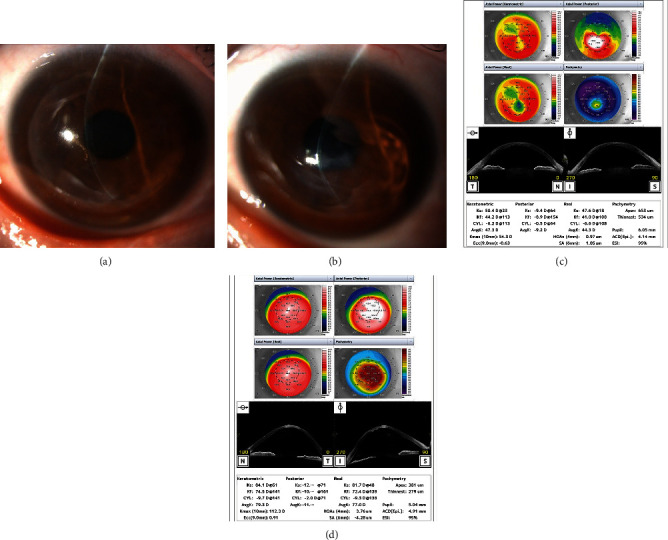
Slit-lamp photograph obtained 30 years after epikeratophakia. (a) Right eye (operated). (b), Left eye (nonoperated). The right eye shows an epikeratophakia graft (central area transparent), and the left eye has a central protrusion; opacity is observed after acute hydrops. (c), Right eye. (d), Left eye. Anterior-segment optical coherence tomography (AS-OCT) findings 30 years after epikeratophakia. The left eye is remarkably protruded compared with the right eye.

**Figure 2:**
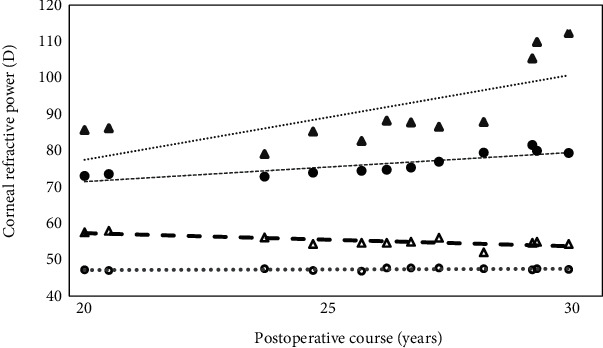
Average keratometry (AvgK) and maximum keratometry (K_max_) changes on AS-OCT 20–30 years after epikeratophakia. 〇 AvgK (right); ● AvgK (left); △ K_max_ (right); ▲ K_max_ (left). Progression has stopped in the right eye (operated) but continued in the left eye (nonoperated).

During the follow-up period, the condition in the nonoperated left eye continued to progress, resulting in an episode of acute hydrops 9 years and 10 months after surgery. As a result, visual acuity in the left eye deteriorated to RV = 0.1 (1.0×−13.0 D = cyl − 4.5 DA × d115°) and LV = 20 cm/n.d. Although acute hydrops resolved spontaneously, the keratoconus had progressed in the left eye (Figures 1(a) and 1(b)). Visual acuity 30 years after surgery was as follows: RV = (1.0 × HCL) and LV = (0.4 × HCL). Anterior-segment optical coherence tomography (AS-OCT) became available 20 years after surgery. The AS-OCT measurements taken 30 years after the operation showed an average keratometry (AvgK) and a maximum keratometry (*K*_max_) of 47.3 and 54.3 D, respectively, in the right eye and 79.5 and 112.3 D, respectively, in the left eye. The central and smallest corneal thicknesses in the right eye were 653 and 534 *μ*m, respectively, while those in the left eye were 381 and 279 *μ*m, respectively (Figures 1(c) and 1(d)). However, it is noteworthy that these measurements were taken 30 years after the epikeratophakia procedure. The changes in AvgK and *K*_max_ from 20 to 30 years after the surgery were +0.5 and −0.3 D in the right eye, respectively, and +4.9 and 29.7 D in the left eye, respectively (Figure 2).

## 3. Discussion

In the present case, keratoconus progression halted in the operated eye, and the corneal thickness was maintained even 20 years after the surgery. To the best of our knowledge, the longest follow-up duration of epikeratophakia for the aphakic eye was 28 years, as reported by Kang et al. [4]. Studies by Spitznas et al. [5] and Panda et al. [6] reported follow-up durations of 7 and 10 years, respectively. Both reports showed good results with stable refraction and improved visual acuity. Based on the present case and previously reported long-term results [4–6], epikeratophakia appears to be effective in stabilizing the refraction and halting the progression of keratoconus in the long term. However, since this was a single case report, further studies are required to evaluate the efficacy of epikeratophakia in halting the keratoconus progression.

Although the role of Bowman's membrane in the pathogenesis of keratoconus remains unclear, its thickness is reported to be reduced in keratoconic corneas [7]. The Bowman layer transplantation [8], in addition to allograft or xenograft corneal lenticule implantation [9, 10], has been shown to halt the progression of keratoconus. In the case of epikeratophakia, the epithelium of the lenticel is removed; however, Bowman's membrane is preserved for transplantation. Therefore, transplanting a healthy Bowman layer may be associated with arresting the progression of the disease. Additionally, in a previous case, corneal cross-linking (CXL) was performed 1 month after epikeratophakia using a lenticel of small incision lenticule extraction, and good results were achieved 3 years after surgery [11]. In this case, the smallest corneal thickness of the nonoperated contralateral eye was 279 *μ*m, and this was not suitable for CXL. In the contralateral eye for which DALK is not indicated owing to a history of acute hydrops, epikeratophakia, as well as allograft or xenograft lenticule transplantation, may be considered as a treatment option before performing penetrating keratoplasty from the viewpoint of preserving the corneal thickness. Furthermore, increased corneal thickness may allow the opportunity for CXL to be indicated again. In the operated eye, the postoperative corneal thickness remained constant at 635 *μ*m in the central area and at 529 *μ*m in the thinnest area, even after 30 years.

In conclusion, epikeratophakia can be considered a viable treatment option for restoring corneal thickness in patients with keratoconus that has progressed beyond the level of indication for CXL. However, randomized controlled trials are required to verify this conclusion.

## Data Availability

The data that support the findings of this study are available upon reasonable request from the corresponding author. The data are not publicly available because they contain information that could compromise the privacy of the patient.
